# Immortalization-upregulated protein promotes pancreatic cancer progression by regulating NPM1/FHL1-mediated cell-cycle-checkpoint protein activity

**DOI:** 10.1007/s10565-022-09695-4

**Published:** 2022-02-10

**Authors:** Qiankun Luo, Yanfeng Pan, Qiang Fu, Xu Zhang, Shuai Zhou, Pengfei Yu, Huiyuan Tian, Pan Liu, Song Chen, Hongwei Zhang, Tao Qin

**Affiliations:** 1 Department of Hepatobilliary and Pancreatic Surgery, Zhengzhou University People’s Hospital, Henan Provincial People’s Hospital, No.7, Weiwu Rd., Jinshui District, Zhengzhou, 450003 Henan China; 2 Department of Infection Disease, the First Affiliated Hospital of Zhengzhou University, No. 1, Jianshe East Rd. Erqi District, Zhengzhou, 450003 Henan China; 3 Translational Research Institute, Henan Provincial People’s Hospital, Zhengzhou University People’s Hospital, Academy of Medical Sciences, Zhengzhou University, Zhengzhou, 450003 Henan China; 4 Department of Research and Discipline Development, Henan Provincial People’s Hospital, Zhengzhou University People’s Hospital, No.7, Weiwu Rd., Jinshui District, Zhengzhou, 450003 Henan China; 5 Henan University People’s Hospital, No.7, Weiwu Rd., Jinshui District, Zhengzhou, 450003 Henan China

**Keywords:** IMUP, Pancreatic cancer, Cell cycle, S phase

## Abstract

Immortalization-upregulated protein (IMUP) plays a vital role in cell proliferation and tumor progression. However, its role in pancreatic ductal adenocarcinoma (PDAC) remains unclear. Here, we select *IMUP* as an alternative gene based on GeneChip analysis of clinical PDAC tissues and transcriptome data from The Cancer Genome Atlas. *IMUP* expression is upregulated in PDAC tumor tissues. Moreover, high *IMUP* expression correlates with poor prognosis, while *IMUP* depletion inhibits PDAC cell proliferation and colony formation capacity in vitro, and decreases xenograft tumor growth in vivo. *IMUP* downregulation leads to cell-cycle arrest in the S phase. *IMUP* knockdown increases the expression of four-and-a-half LIM domain protein 1 (FHL1), which regulates the phosphorylation of cell division cycle 25A (CDC25A) by cycle checkpoint kinase 1 (CHK1) and promotes cytoplasmic distribution of CDC25A by interaction with 14–3-3ξ. Furthermore, *FHL1* knockdown restores the effects induced by *IMUP* depletion. Liquid chromatography tandem mass spectrometry and immunoprecipitation analysis further show that *IMUP* interacts directly with nucleophosmin (NPM1) and enhances its stability. DNA methylation sequencing shows that *FHL1* promoter methylation decreases when *IMUP* is downregulated. Overexpression of *NPM1* can increase the methylation level of *FHL1*, thereby decreasing its expression. Our study provides a novel perspective on IMUP/NPM1/FHL1-mediated cell-cycle arrest by regulating CDC25A phosphorylation in PDAC. These findings may provide a new therapeutic target for PDAC.

**Graphical abstract:**

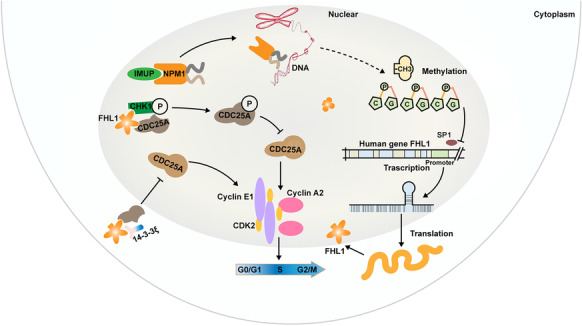

**Supplementary Information:**

The online version contains supplementary material available at 10.1007/s10565-022-09695-4.

## Introduction

Pancreatic ductal adenocarcinoma (PDAC) is a malignant tumor with poor prognosis (Siegel et al. 2019). Although various hallmark capacities of cancer have been recognized (Hanahan and Weinberg, 2011), no effective pharmacotherapeutic or chemotherapeutic strategies exist for PDAC. Among the ten cancer hallmarks, one of the most important is sustaining cell proliferation capability (Hanahan and Weinberg, 2011). The abnormal regulation of cell-cycle checkpoints greatly contributes to malignant cell proliferation. DNA damage caused by ionizing radiation, chemicals, and drugs typically induces cell-cycle checkpoint arrest, thus, providing the opportunity for increased DNA repair (Murakami and Nurse, 2000). However, cancer cells acquire the ability to circumvent cell-cycle checkpoints by activating intracellular signaling pathways, thereby, facilitating their continual grow. In earlier studies, many researchers focused on the regulation of G1 and G2 checkpoints as prominent cell-cycle arrests were more readily observed in the G1 and G2 phases. Meanwhile, the S-phase checkpoint has a more significant role in maintaining genetic stability than either G1 or G2 phases (Bartek et al. 2004).

Immortalization-upregulated protein (IMUP) was first identified in SV40-immortalized fibroblasts and includes two isoforms, namely, IMUP-1 and IMUP-2 (Kim et al. 2000). IMUP is reportedly upregulated in many cancer tissues and cell lines, while its overexpression is associated with tumorigenicity (Ryoo et al. 2006) and cell-cycle acceleration (Jeon et al. 2013). Moreover, Qian et al. reported that IMUP is highly expressed in PDAC tumor tissues based on bioinformatic analysis (Qian et al. 2021). However, the function and mechanism of IMUP in PDAC have not yet been characterized.

Four-and-a-half LIM domain protein 1 (FHL1) is downregulated in many cancers, including gastric (Asada et al. 2013), breast (Ding et al. 2011), and lung cancers (Wang et al. 2018a, b, c). FHL1 has been confirmed as a tumor suppressor that inhibits cell growth, invasion, and cancer progression. Xu et al. reported that FHL1 interacts with cell division cycle 25 (CDC25), cell-cycle checkpoint kinase (CHK) 2, and 14–3-3ξ in HeLa and MCF-7 cell lines, all of which represent key cell-cycle checkpoint proteins that participate in cell-cycle progression regulation (Xu et al. 2017). Remarkably, the downregulation of *FHL1* in gastric (Asada et al. 2013) and liver cancers (Wang et al. 2017) is associated with promoter methylation. Nucleophosmin (NPM1), a nucleocytoplasmic shuttling protein, has a sandwich-like structure that allows it to form a histone complex, thus regulating diverse cell biological processes, including epigenetic modification (Holmberg Olausson et al. 2015), cell-cycle progression, chromatin remodeling, and transcription regulation (Karimi Dermani et al. 2021). Moreover, NPM1 reportedly plays an important role in various solid tumors (Qin et al. 2020; Xu et al. 2014). However, the potential molecular mechanisms of FHL1 and NPM1 in PDAC have not yet been elucidated.

Here, we discovered that IMUP is upregulated in PDAC tumors and has a negative impact on the survival rates of patients. Downregulation of IMUP inhibits cell growth in vivo and in vitro via cell-cycle arrest at the S phase, which is related to increased expression of FHL1. In addition, we confirmed that IMUP enhances NPM1 stability via direct binding. IMUP inhibits the transcription of *FHL1* through NPM1-mediated epigenetic modification. Our results provide novel insights into the cell-cycle checkpoint mechanisms regulated by IMUP, which may contribute to the future exploration of biological markers and therapeutic targets for PDAC.

## Materials and methods

### GeneChip analysis and data analysis of The Cancer Genome Atlas (TCGA)

Total RNA was isolated from 20 samples (collected from Henan Provincial People’s Hospital), including ten tumor tissues and ten adjacent normal tissues, using an mirVana™ RNA isolation kit (AM1561, Thermo Fisher, USA). RNA clean-up was performed using RNasey Mini Kit (Qiagen p/n 74,104). Quantification and quality control were achieved by NanoDrop ND-2100 (ThermoFisher) and Agilent Bioanalyzer 2100 (Agilent Technologies, Palo Alto, USA). cDNA synthesis and biotin labeling of cRNA were performed using One-Cycle Target Labeling and Control Reagents (Affymetrix, Santa Clara, USA) and the GeneChip™ Hybridization, Wash and Stain Kit (Affymetrix) following the manufacturer’s protocol. The purified and fragmented labeled cRNAs were hybridized onto the Affymetrix PrimeView™ Human Gene Expression microarray. After washing and staining, the arrays were scanned and analyzed using Affymetrix Scanner 3000 (Affymetrix). GeneChip Command Console Software (version 4.0, Affymetrix) and Genespring software (version 14.9, Agilent Technologies) were used to analyze the transcriptome data. Differentially expressed genes (DEGs) from TCGA were analyzed using DESeq2 package within R language (version 3.4.3).

### Human tissues and immunohistochemistry (IHC)

Tumor tissues and adjacent normal tissues were collected from 57 surgically resected PDAC patients at Henan Provincial People’s Hospital for GeneChip analysis, western blotting (WB), and IHC analysis. The clinical stage of the disease was defined according to the 8th edition of the American Joint Committee on Cancer classification. IHC was performed according to the protocol described in our previous study (Zhang et al. 2020). Antibodies against IMUP (1:100; #ab221063) from Abcam (Cambridge, UK) and FHL1 (1:50; #10,991–1-AP) from Proteintech (Wuhan, China) were used for IHC to verify their expression in PDAC and xenograft tumor tissues. The IHC scores were produced by multiplying the proportion of positive cells by the dyeing intensity (pale yellow, 1; brownish yellow, 2; brown, 3). The expression level was defined as high or low when the score was ≥ 1 or < 1, respectively. All the human tissues were de-identified before analysis.

### Cell culture, cell proliferation, and colony formation assays

Human immortalized pancreatic ductal epithelial cells (HPDE), HEK293T cells, and human PDAC cell lines, including BxPC-3, SW1990, and PANC-1 cells, were purchased from the American Tissue Type Culture Collection. MIA paca-2 cells were purchased from the National Collection of Authenticated Cell Cultures (Shanghai, China). The results of mycoplasma contamination tests were negative. Cells were cultured in Dulbecco’s modified Eagle’s medium (Invitrogen, CA, USA) containing 10% fetal bovine serum (Biological Industries, Israel). The concentration of DNA methyltransferase inhibitor 5′-aza-2′-deoxy-cytidin (5′-aza) (Sigma, St. Louis, MO, USA) used in the medium was 0.5 μm. In the cycloheximide (CHX) (Invitrogen) assays, cells were treated with 100 μg/mL CHX for 0, 12, 24, and 48 h to block protein synthesis. Cell Counting Kit-8 and colony formation assays were performed as previously described (Xia et al. 2020).

### Flow cytometry

The cell cycle was detected using propidium iodide and RNase staining solution according to the manufacturer’s protocol (Beyotime, C1052). Flow cytometry data were obtained using a BD FACSAria™ III Cell sorter (USA) and analyzed using ModFit version LT4.1.

### Mouse xenograft

Five-week-old male BALB/c nude mice were bred by Vital River (Beijing, China). All mice were raised and treated following the guidelines of the Zhengzhou University Laboratory Animal Care Committee (ZZU-LAC20210416[07]). Each mouse was injected in the right forelimb armpit with 1 × 10^7^ BxPC-3 cells, which were suspended in 100 μL phosphate buffered saline. Calipers were used to measure the tumor size every 2 to 3 days. The formula used to compute tumor volume was: π/6 × length × width × height. Tumor masses were subsequently excised, weighed, and stored in a tissue stabilizer (Vazyme, Nanjing, China) for IHC or WB.

### Plasmids, siRNAs, and lentivirus

siRNAs were purchased from RiboBio (Guangzhou, China). To construct IMUP-, FHL1-, or NPM1-expressing plasmids, we amplified and inserted IMUP, FHL1, or NPM1 cDNA into pCDNA3.1 (Invitrogen) for overexpression with a GFP- or Flag-tag. Lentivirus small hairpin RNAs (shRNAs) targeting IMUP and FHL1 were selected from Dharmacon™ GIPZ™ Lentiviral shRNA Library (Cambridge, UK). HEK293T cells co-transfected with lentivirus vectors, gag/pol, VSV-G, and REV were used to produce lentiviruses. After 48 h, the lentiviruses were collected and added to the medium of BxPC-3 or SW1990 cells with polybrene (1:1000; Beyotime, Wuhan, China). The target sequences of the siRNAs and shRNAs are shown in Table S1.

### RNA sequencing (RNA-seq) analysis

BxPC-3 cells were transfected with three different IMUP siRNAs and normal control siRNA (si-NC) for 36 h. Total RNA was extracted from cells using Trizol (Invitrogen) and purified using EpicentreRibo-Zero rRNA Removal Kit (Illumina, USA). Subsequently, a cDNA library was constructed using NEBNext® Ultra™ RNA Library Prep Kit for Illumina® (NEB, USA). RNA-seq was performed on a HiSeq3000 platform (RiboBio). HISAT2 version 2.1.0 was used to align the clean reads. Differential expression was assessed by DEseq R packages (version 1.30.0). DEGs between si-NC and si-IMUP were selected based on fold changes ≥ 2 and adjusted *P* value ≤ 0.05. All the DEGs were used for heat map analysis and Kyoto Encyclopedia of Genes and Genomes enrichment analysis (http://www.genome.jp/).

### WB, immunoprecipitation (IP), and liquid chromatography tandem mass spectrometry (LC/MS)

Antibodies against IMUP (1:500; #ab228823), pCDC25A (Phospho S124; 1:1000; #ab156574), pCHK1 (1:500; ab79758), from Abcam; cyclin-dependent kinase 2 (CDK2) (1:1000; #AF6237) from Affinity Biosciences (OH, USA); Cyclin A2 (1:1000; #BF683) and Cyclin E1 (1:1000; #HE12) from Cell Signaling Technology (Danvers, MA, USA); and FHL1 (1:500; #10,991–1-AP), CDC25A (1:1000; #55,031–1-AP), CHK1 (1:1000; #25,887–1-AP), CDC25C (1:1000; #16,485–1-AP), 14–3-3ξ (1:1000; #11,648–2-AP), SP1 (1:1000; #21,962–1-AP), and NPM1 (1:1000; #10,306–1-AP) from Proteintech were used for WB assays.

Antibodies against IMUP (1:100; #ab228821) from Abcam; GFP-Trap® Magnetic Agarose (20 μL per reaction; #gtma-100) from Proteintech; and Pierce™ anti-DYKDDDDK Magnetic Agarose (20 μL per reaction; #A36798) from Invitrogen were used for IP to detect protein interactions. For LC/MS, immunocomplexes from GFP-Trap® Magnetic Agarose, anti-IMUP agarose, or anti-DYKDDDDK magnetic agarose were separated by sodium dodecyl sulfate polyacrylamide gel electrophoresis. Subsequently, the candidate bands were cut and identified by LC/MS.

### DNA methylation analysis

BxPC-3 cells were transfected with control shRNA, IMUP-shRNA, or co-transfected with GFP-tagged NPM1 vector. The DNeasy Tissue Kit (QIAGEN) was used to extract total DNA. Genomic DNA was bisulfite converted using the EpiTect Bisulfite Kit (QIAGEN) and amplified by PCR using FHL1 promoter-specific primers (fragment 1: forward GGGTTTAGTAAATTGAATGTTGAGT, reverse CCCCATCCATAATCCCAATAC; fragment 2: forward TGGTTTTTTAGGGTTGGGTAT, reverse TCCACCCCACTACCTCTTAA). Pyrosequencing was performed on the PyroMark Q48 Autoprep (QIAGEN) according to the manufacturer’s instructions.

### Quantitative real-time PCR

TRIzol reagent (Beyotime), HiScript RT SuperMix (Vazyme), and PowerUpTM SYBRTM Green Master Mix (ThermoFisher) were used for RNA extraction, reverse transcription, and quantitative real-time PCR (RT-qPCR) as previously described with the primers listed in Table S2 (Xia et al. 2020).

### Chromatin immunoprecipitation assay (ChIP)

Antibodies against SP1 (1:50; #21,962–1-AP) from Proteintech and ChIP Assay Kit (P2078) from Beyotime were used for ChIP assay according to the manufacturer’s instructions. Two SP1-binding sites of *FHL1* promoter region were determined by RT-PCR. Details of the primers used are provided in supplementary Table S2. The *FHL1* upstream and actin promoter were employed as negative controls.

### Statistical analysis

GraphPad Prism 5.0 and SPSS Statistics 22.0 were applied for data analysis. Comparisons between two groups were assessed using unpaired two-tailed Student’s *t* test or paired-samples *t* test. The Mann–Whitney *U* test or Wilcoxon signed rank test was used for non-normal distribution analysis. The correlation of two variables was determined by the Pearson coefficient. The overall survival (OS) and disease-free survival (DFS) curves of patients were plotted using the Kaplan–Meier method, and statistical differences were verified by a Log-rank test. All statistical tests were two-tailed, and *P* < 0.05 was considered statistically significant.

## Results

### IMUP is upregulated in PDAC and associated with poor prognosis

**Fig. 1:**
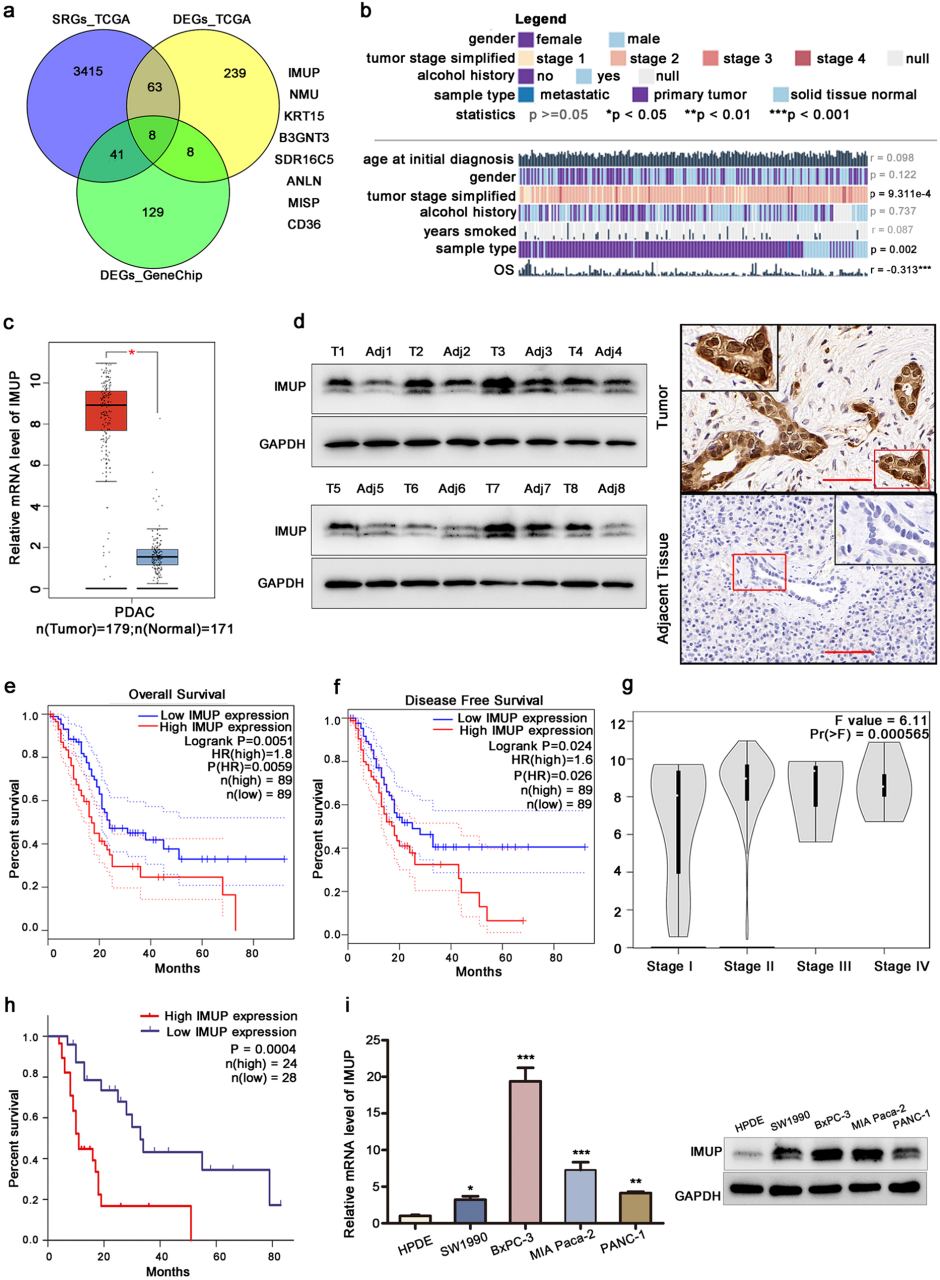
IMUP is upregulated in PDAC and correlates with poor prognosis.** a** IMUP was selected from the intersection of differentially expressed genes (DEGs) from GeneChip, DEGs from TCGA, and survival-related genes (SRGs) from TCGA. **b** *IMUP* expression differs between different simplified tumor stages (*P* < 0.0001) and is negatively correlated with overall survival (OS). No differences were observed between the general characteristics of patients, including age, gender, years smoked, alcohol history (*P* > 0.05). **c** *IMUP* mRNA level analyzed by GEPIA. **d** IMUP protein abundance in tumors and adjacent tissues from PDAC patients by WB tests. Representative immunohistochemical staining of IMUP in PDAC tumor tissues and adjacent tissues. Scale bar: 100 μm. **e**, **f**, **g** Kaplan–Meier analysis of OS and disease-free survival (DFS), and clinical stage analysis of TCGA data. **h** Kaplan–Meier analysis of OS of our clinical data. **i** mRNA and protein level of IMUP in BxPC-3, SW1990, PANC-1, and human immortalized pancreatic ductal epithelial cells (HPDE). An unpaired Student’s *t* test is used to perform statistical analysis

We detected the transcriptomics of tumor and adjacent normal tissues of ten paired primary PDAC patients by microarray scanning using GeneChip. DEGs between tumor and adjacent tissues were analyzed (Fig. S1a, b). In addition, we obtained DEGs and survival-related genes (SRGs) from the TCGA database (http://www.cbioportal.org/index.do). The intersecting DEGs between GeneChip (fold change > 4), TCGA, and SRGs identified *IMUP* as the most notable gene (Fig. 1a).

The data from the MEXPRESS online tool indicated that tumor stage and OS of PDAC patients were associated with *IMUP* expression (Fig. 1b) (Koch et al. 2019). The general characteristics of patients, including age, sex, years smoked, and alcohol history, were not significantly different in terms of *IMUP* expression (*P* > 0.05; Fig. 1b). Consistent with these results, bioinformatics analysis of data from TCGA and GTEx databases by GEPIA demonstrated that the mRNA level of *IMUP* was upregulated in tumors (Fig. 1c) (Tang et al. 2019).

Protein levels were investigated in PDAC tumor tissues and adjacent normal tissues using WB and IHC. Results showed that the IMUP abundance in tumor tissues was higher than that in adjacent tissues (Fig. 1d, Fig. S2a). Moreover, data from GEPIA demonstrated that patients with high IMUP abundance had a poor OS, DFS, and an advanced clinical stage (Fig. 1e–g) (Tang et al. 2019), which was consistent with the results of Kaplan–Meier analysis of our clinical tissues (*P* < 0.001) (Fig. 1h). Univariate and multivariate Cox regression analyses confirmed that high IMUP abundance was a significant predictor of OS (hazard ratio = 3.695, *P* < 0.001; Table S4). Finally, we verified that IMUP levels in PDAC cell lines, including BxPC-3, SW1990, MIA Paca-2, and PANC-1, were significantly elevated in comparison with those in HPDE cells (Fig. 1i). Collectively, these data suggest that IMUP is upregulated in PDAC and negatively affects patient prognosis. Hence, IMUP may play a critical role in PDAC progression.

### *IMUP knockdown inhibits cell growth *in vitro* and *in vivo

**Fig. 2:**
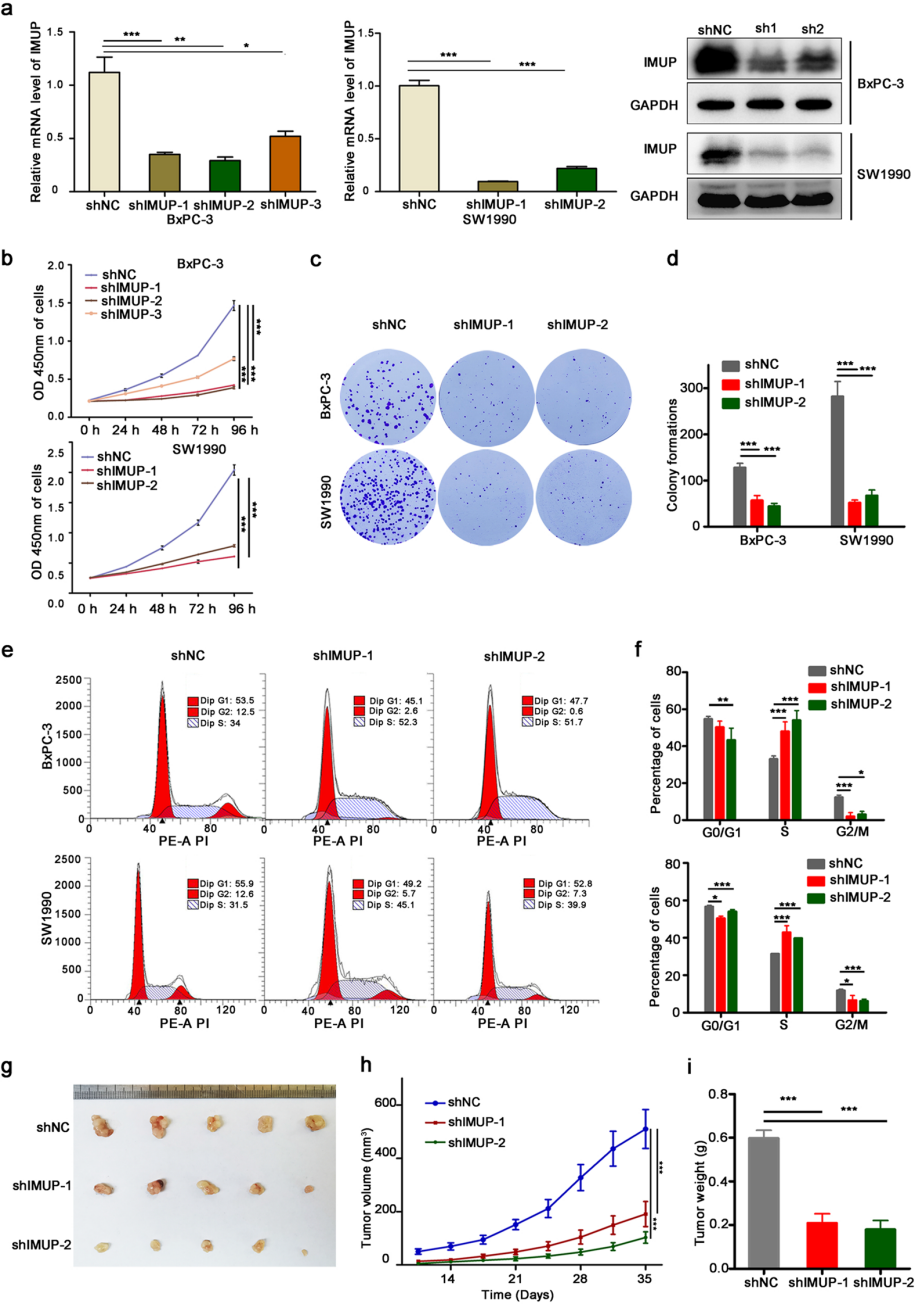
Knockdown of** IMUP** inhibits cell growth and tumorigenicity. BxPC-3 and SW1990 cells infected with normal control shRNA (shNC), IMUP-shRNA1 (sh1), sh2, and sh3 were used to analyze **a** IMUP expression by RT-qPCR and WB, **b** proliferation by cell Counting Kit-8, **c** colony formation capability by colony formation assay, **e** cell cycle by flow cytometry, and **g** tumorigenicity by mice xenograft (*n* = 5/group; male). GAPDH was used as loading control for WB. Unpaired Student’s *t* test was used to analyze **a** RT-qPCR data and **i** weight. **d** Colony formation, **f** cell cycle, and **h** xenograft tumor volume were analyzed by two-way ANOVA test. *P < 0.05, **P < 0.01, ***P < 0.0001

Three shRNAs were used to interfere with *IMUP* expression in BxPC-3 and SW1990 cells. The mRNA and protein levels of IMUP were markedly depleted by shRNA-1 and shRNA-2 (Fig. 2a), and *IMUP* knockdown significantly inhibited the proliferative and colony formation capacity of BxPC-3 and SW1990 cells (Fig. 2b–d). Flow cytometry results revealed that the cells were blocked at the S phase by *IMUP* depletion (Fig. 2e, f). Moreover, *IMUP* depletion inhibited tumor xenograft formation of BxPC-3 cells in BALB/c nude mice (Fig. 2g). The volume and weight of IMUP-sh1 and IMUP-sh2 xenograft tumors also decreased significantly in comparison with the normal control group (shNC) xenograft tumors (Fig. 2h, i). IHC was used to assess *IMUP* downregulation in the xenograft tumors (Fig. S2b). Results indicated that *IMUP* knockdown significantly suppressed tumor growth in vitro and in vivo, consequently confirming the potent role of IMUP in PDAC tumorigenicity.

### *IMUP regulates cyclin A2/cyclin E1/CDK2 proteins *via* FHL1*

**Fig. 3:**
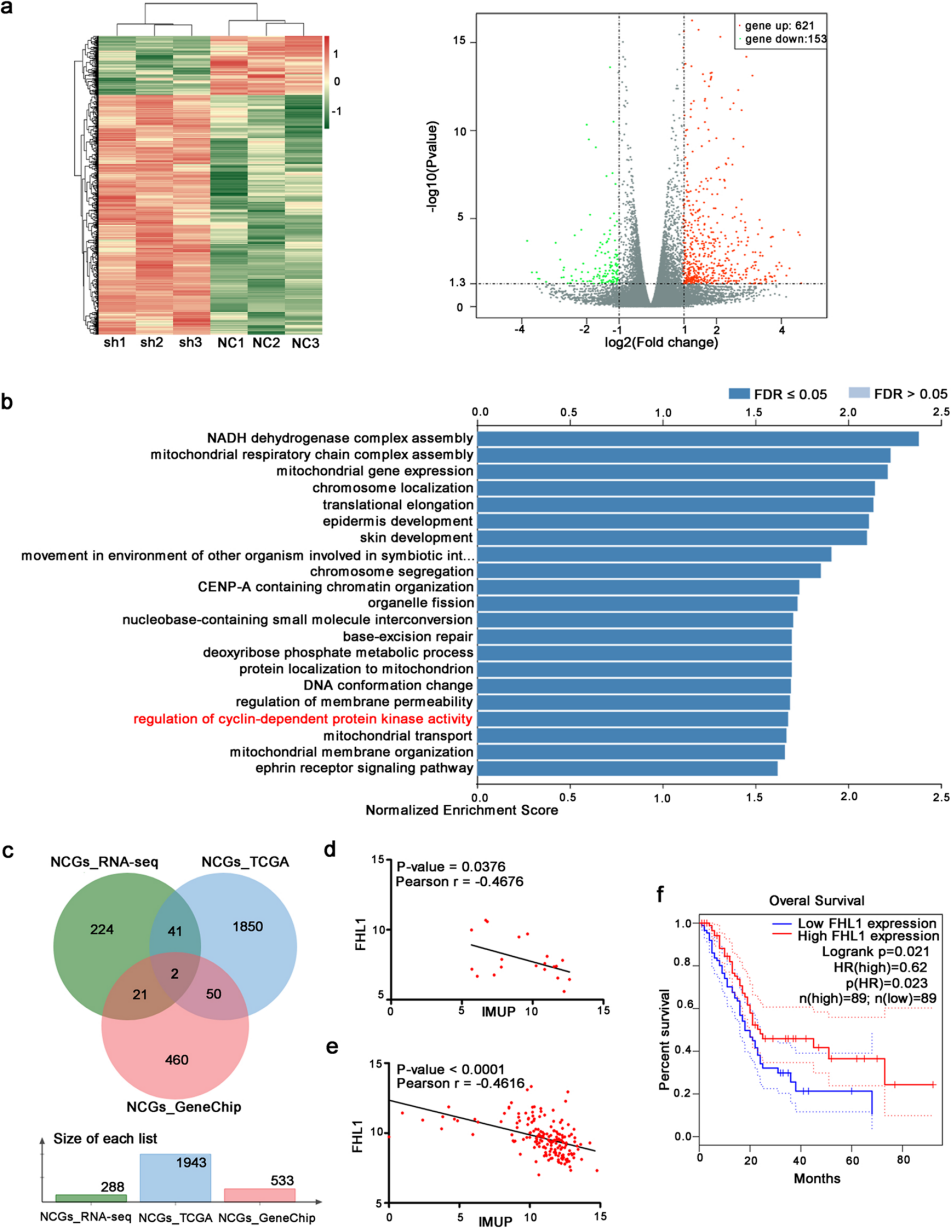
Exploring downstream gene regulated by IMUP.** a** Heatmap and volcano plot analyzed using RNA-seq of BxPC-3 cells infected with shNC, IMUP-sh1, sh2, and sh3. **b** Gene ontology analysis of IMUP-correlated genes from TCGA database by LinkedOmics. **c** The intersection of negatively correlated genes (NCGs) from RNA-seq (fold-change > 2), NCGs from GeneChip, and NCGs from TCGA. **d**, **e** Pearson correlation of FHL1 and IMUP expression according to **d** the results of GeneChip and **e** TCGA data. **f** Kaplan–Meier analysis of OS by FHL1 expression data from TCGA

To investigate the mechanisms of IMUP in cell-cycle arrest, we performed RNA-seq for BxPC-3 cells with or without *IMUP* knockdown. A total of 621 genes were differentially upregulated, while 153 genes were downregulated following *IMUP* depletion (Fig. 3a). In addition, we analyzed IMUP-correlated genes from the TCGA database using LinkedOmics (Vasaikar et al. 2018). Gene Ontology (GO) analysis results showed that IMUP-associated genes were enriched in regulation of cyclin-dependent protein kinase activity (Fig. 3b). The positively and negatively correlated genes (PCGs and NCGs, respectively) associated with IMUP were analyzed using the results from GeneChip, RNA-seq (fold-change > 2), and the TCGA database. The intersection of PCGs from them provided wingless-type MMTV integration site family member 10A (WNT10A) (Fig. S3a), whereas that of NCGs from them provided G protein subunit gamma 2 and FHL1 (Fig. 3c), which might be regulated by IMUP. Among these DEGs, *FHL1* is reportedly associated with negative regulation of cell growth. The data from GeneChip and TCGA verified that *FHL1* expression was significantly negatively correlated with IMUP (Fig. 3d, e). Moreover, high expression of *FHL1* correlated with longer OS (*P* = 0.023) in patients with PDAC (Fig. 3f) (Tang et al. 2019). Thus, we hypothesized that FHL1 may mediate the inhibition of tumor growth induced by IMUP depletion.

**Fig. 4:**
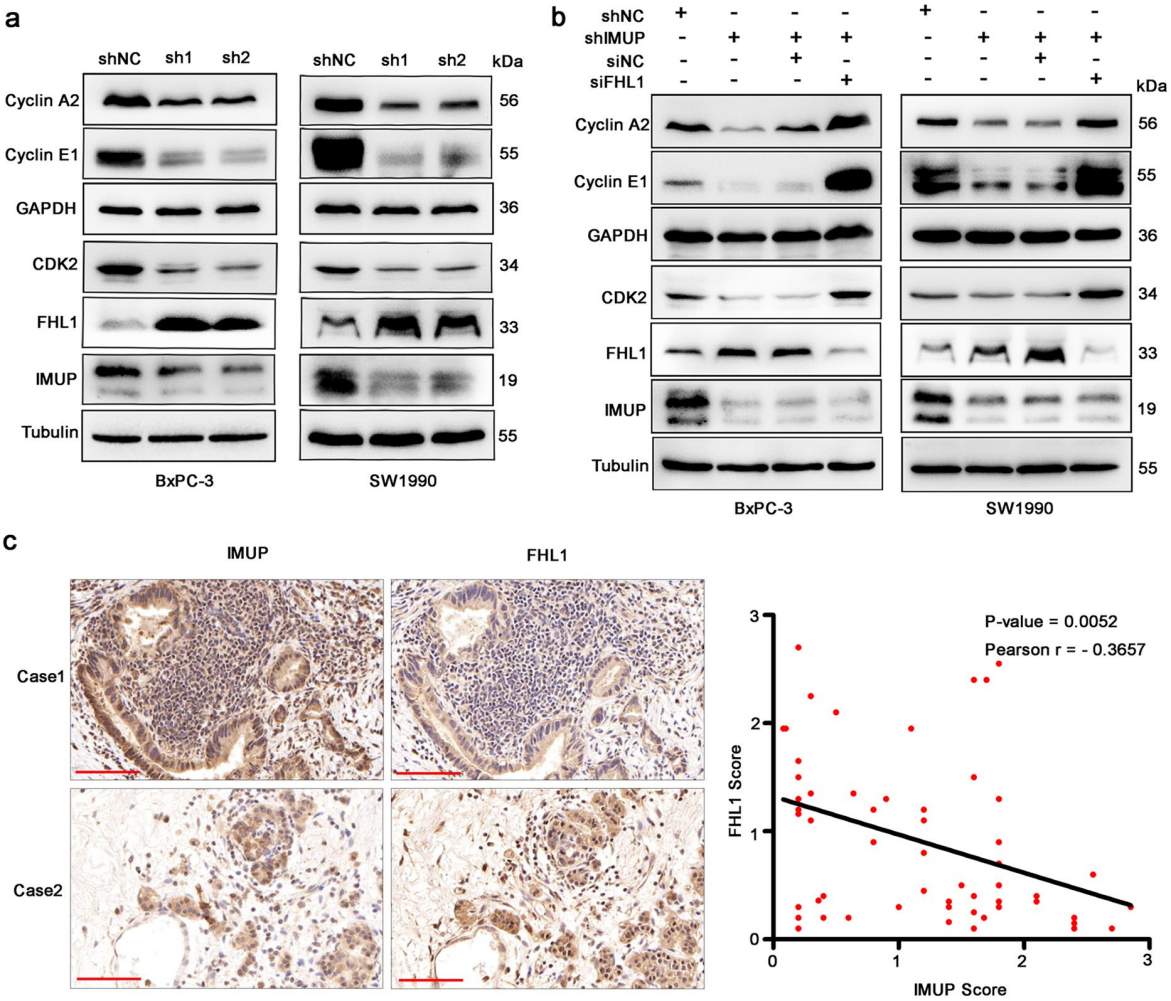
IMUP regulates cyclin A2/cyclin E1/CDK2 proteins via FHL1.** a** WB analysis of BxPC-3 and SW1990 cells infected with lentivirus carrying IMUP-sh1 or sh2. GAPDH and tubulin were used as a loading control. **b** WB analysis of BxPC-3 and SW1990 cells infected with lentivirus carrying IMUP-sh or co-infected with IMUP-sh and FHL1-siRNA. **c** Representative IHC staining of IMUP and FHL1 in 57 human PDAC samples. Case 1 and case 2 were two representative specimens analyzed as high and low expression of IMUP, respectively. (case 1, high IMUP and low FHL1; Case 2, low IMUP and high FHL1). Scale bars: 100 μm. Correlation between IMUP and FHL1 analyzed by Pearson correlation tests

The WB results showed that knockdown of *IMUP* decreased the abundance of cyclin A2, cyclin E1, and CDK2 in BxPC-3, SW1990, PANC-1, and MIA Paca-2 cells, which are the key protein kinases of the S phase, and increased the mRNA and protein levels of FHL1 (Fig. 4a; Fig. S3b and c). The silencing of *FHL1* via siRNA restored the protein inhibition induced by IMUP-shRNA (Fig. 4b). In addition, the results of PDAC tumor tissue IHC revealed that high expression of IMUP was associated with a low level of FHL1, while Pearson’s correlation analysis suggested that their IHC scores were negatively correlated (Fig. 4c). These results confirm that IMUP regulates cell-cycle protein activity of PDAC cells by FHL1.

### Knockdown of FHL1 rescues the phenotype inhibited by IMUP depletion

**Fig. 5:**
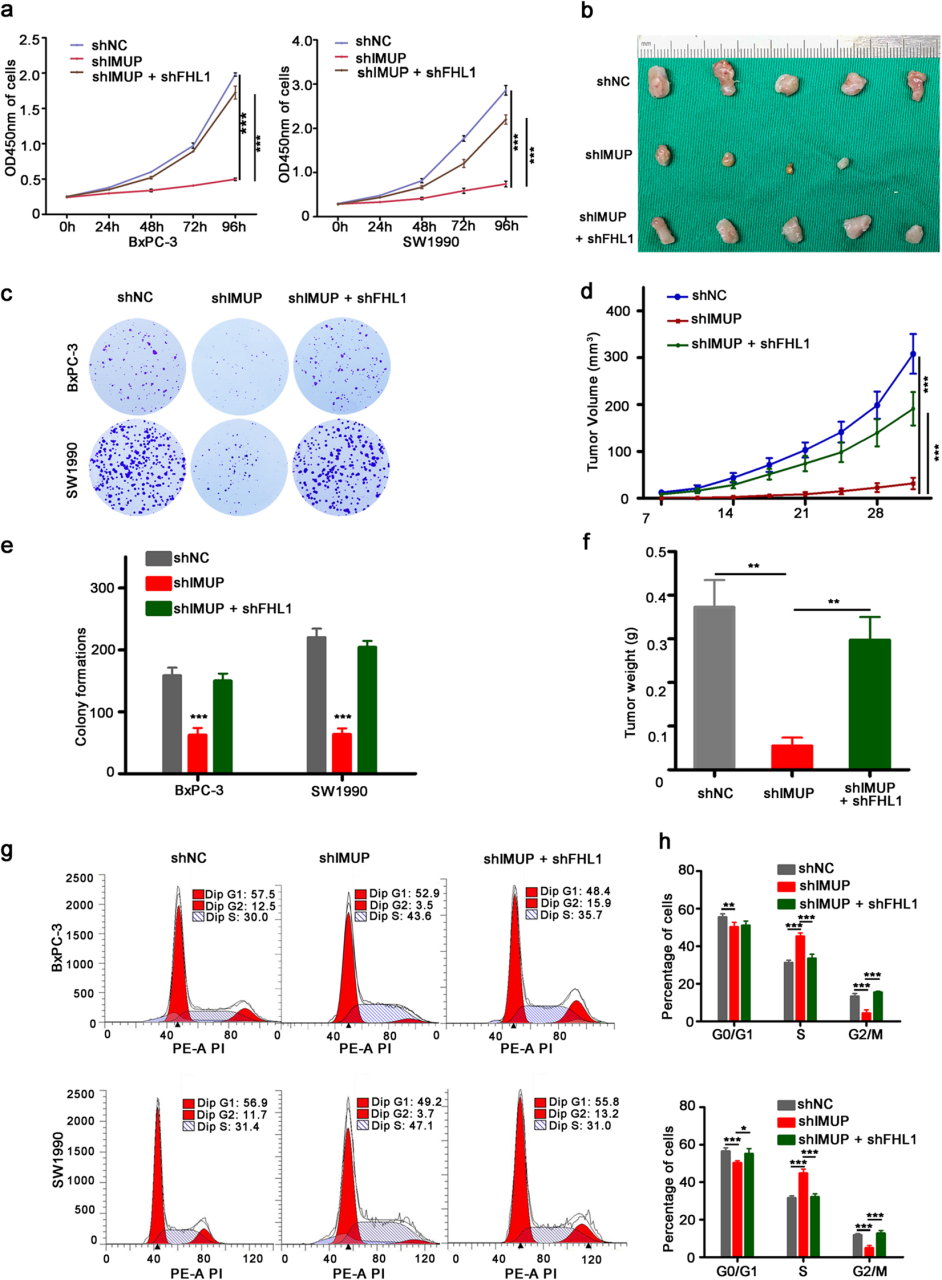
Knockdown of *FHL1 *rescues the phenotype inhibited by IMUP depletion.** a**, **b**, **c**, **g** BxPC-3 and SW1990 cells infected with shNC or IMUP-sh, or co-infected with IMUP-sh and FHL1-sh were used to analyze the **a** proliferation by cell Counting Kit-8, **b** tumorigenicity by mice xenograft (*n* = 5/group; male), **c** colony formation capability by colony formation assay, and **g** cell cycle by flow cytometry. **d** Xenograft tumor volume, **e** colony formation, and **h** cell cycle were analyzed by two-way ANOVA test. **f** Tumor weight was analyzed by unpaired Student’s *t* test. *P < 0.05, **P < 0.01, ***P < 0.0001

To determine whether FHL1 is a potential target of IMUP that promotes tumor growth, *FHL1*-shRNA was co-transfected with IMUP-shRNA into BxPC-3 and SW1990 cells. Results showed that *FHL1*-shRNA restored cell proliferation, colony formation, S phase arrest, and tumor xenograft growth in mice, which were inhibited by *IMUP* knockdown (Fig. 5a–c, g). The data showed significant statistical differences among IMUP-shRNA cells, control shRNA cells, and cells co-transfected with IMUP-shRNA and FHL1-shRNA (Fig. 5d–f, h). Thus, we confirmed that IMUP promotes tumor progression of PDAC by regulating FHL1 expression.

### IMUP inhibits the transcription of FHL1 by NPM1-induced promoter methylation

**Fig. 6:**
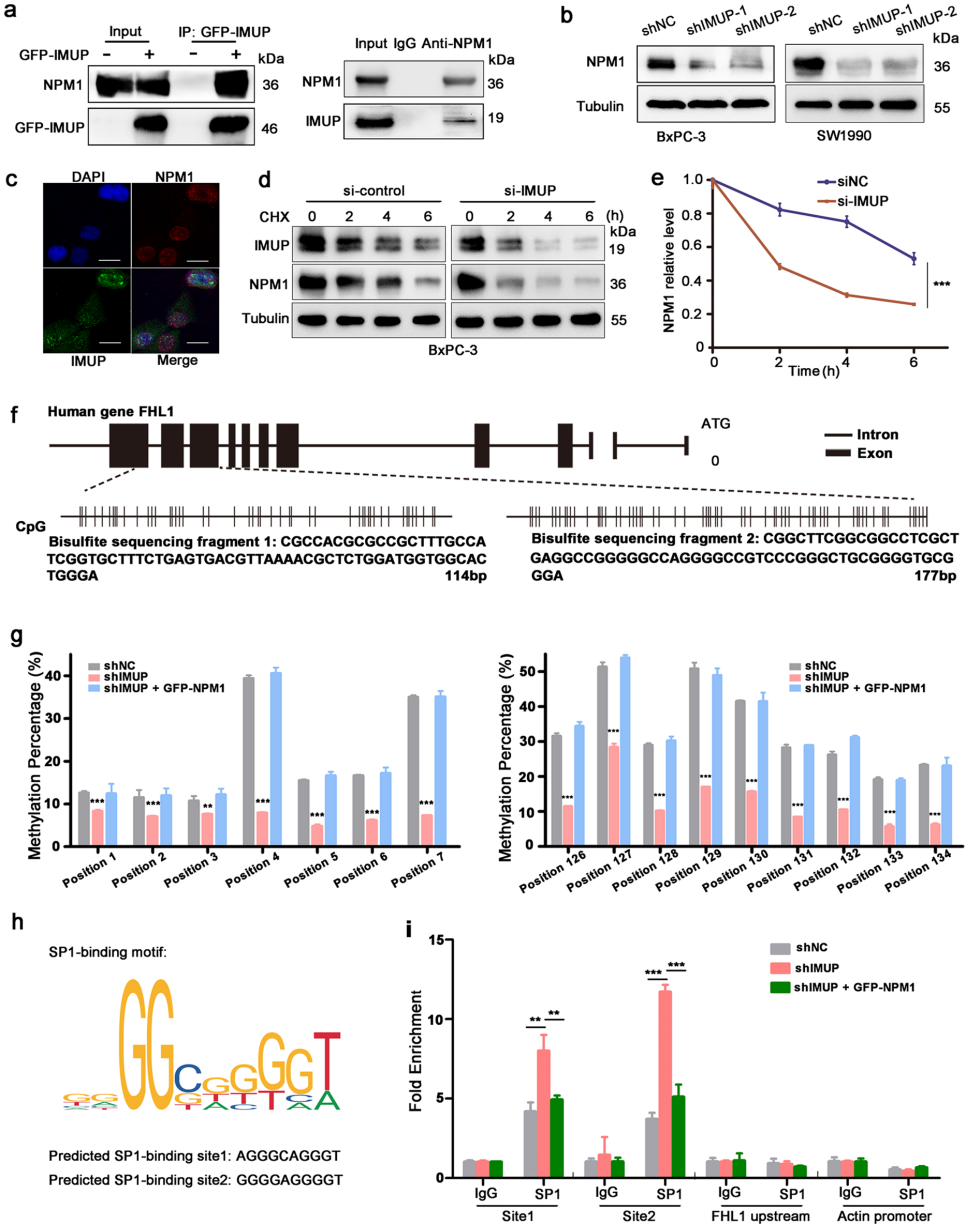
IMUP inhibits** FHL1** transcription by NPM1-induced promoter methylation.** a** BxPC-3 cells were transfected with GFP-tagged IMUP. Exogenous co-immunoprecipitation was performed by GFP beads (left). Endogenous interaction was tested in BxPC-3 cells by anti-NPM1 (right). **b** WB analysis of proteins extracted from BxPC-3 infected with control shRNAs, IMUP sh1, or sh2. **c** Immunofluorescence double-staining of BxPC-3 cells showed the location of IMUP and NLM1. Cells were stained with anti-IMUP (green) and anti-NPM1 (red). The nuclei were stained with DAPI (blue). Scale bars: 50 μm. **d** BxPC-3 cells transfected with *IMUP* or control siRNAs were treated with 100 μg/mL CHX at the indicated time. Proteins were analyzed with anti-IMUP, anti-NPM1, and anti-tubulin. **e** The densitometric quantitation of NPM1 protein at the indicated time was normalized by tubulin. Statistical differences were analyzed by two-tailed Student’s *t* test (***P < 0.0001). **f** Structure of the human FHL1 gene. Dotted lines indicate two exon fragments containing CpG islands and corresponding sequences. **g** The pyrosequencing maps of FHL1 promoter CpG islands. DNA was collected from BxPC-3 cells infected with control shRNAs, IMUP-shRNAs, or co-transfected with IMUP-shRNAs and NPM1 vectors. The methylation rates of *FHL1* promoter CpG islands. Fragment 1 (left histogram) and fragment 2 (right histogram). Statistical differences were analyzed using two-way ANOVA test. ***P < 0.0001. **h** Translational factor SP1-binding motif and putative SP1-binding sequences of *FHL1* promoter. **i** ChIP-qPCR revealed SP1 enrichment on the *FHL1* promoter in BxPC-3 cells infected with either control shRNAs, or IMUP-shRNAs or co-transfected with IMUP-shRNAs and NPM1 vectors. The upstream of *FHL1* promoter and actin promoter were used as negative controls. Statistical differences were analyzed by two-tailed Student’s *t* test (**P < 0.001, ***P < 0.0001)

To determine the mechanism by which IMUP regulates FHL1 expression, GFP-tagged IMUP overexpression and endogenous IMUP were used for IP (Fig. S4a). The intersection of GFP-IMUP-binding proteins and endogenous IMUP-binding proteins strongly supports that NPM1 is a probable target of IMUP according to LC/MS analysis (Table S5). Co-IP assay was further used to assess the direct interaction between IMUP and NPM1 (Fig. 6a). Confocal imaging revealed a partial colocalization of IMUP and NPM1 in PDAC cell nucleus (Fig. 6c).

In addition, we found that knockdown of *IMUP* reduced the protein level of NPM1 (Fig. 6b). However, the protein level of IMUP was not affected by NPM1 depletion induced by NPM1-siRNAs (Fig. S5a). The mRNA of *NPM1* was not affected by *IMUP* knockdown (Fig. S5b). We believe that IMUP sustained NPM1 stability. CHX was used to block protein synthesis in BxPC-3 and SW1990 cells transfected with control and *IMUP* siRNAs. The results showed that NPM1 protein degraded slowly after CHX treatment within 48 h in the control group, whereas *IMUP* knockdown obviously facilitated NPM1 protein degradation (Figs. 6d, e, and S5c). These data indicate that IMUP stabilizes nuclear protein NPM1.

Many studies have reported that FHL1 undergoes epigenetic regulation by promoter methylation in various cancers (Koike et al. 2013), and NPM1 plays an important role in epigenetic regulation (Karimi Dermani et al. 2021). Thus, to determine whether IMUP regulated methylation of the *FHL1* promoter through NPM1, we assessed the promoter methylation status of two promoter exon fragments. Pyrosequencing results confirmed that the methylation of 16 CpG sites decreased significantly in IMUP-depleted BxPC-3 cells (Fig. 6f, g). However, NPM1 overexpression restored promoter methylation. The same phenomenon was observed in SW1990 cells (Fig. S6a). We also analyzed the methylation status in clinical samples. The results indicated that the mean methylation level of 16 CpG sites was obviously elevated in patients with IMUP high expression compared to that in patients with IMUP low expression (Fig. S6b). To investigate whether *FHL1* promoter methylation directly affected its transcription, we identified two putative specific protein 1 (SP1)-binding sites in the *FHL1* methylated promoter region: site1 (AGGGCAGGGT) and site2 (GGGGAGGGGT) (Fig. 6h). ChIP qPCR results revealed that SP1 was enriched at two sites of the *FHL1* promoter. Moreover, *IMUP* knockdown increased SP1 enrichment in *FHL1* promoter. NPM1 overexpression inhibited SP1 binding to the *FHL1* promoter (Fig. 6i). Meanwhile, qPCR demonstrated that *FHL1* expression was inhibited by the NPM1 vector following *IMUP* knockdown (Fig. S7a). However, FHL1 expression was restored to previous levels when the cells infected with shIMUP and GFP-NPM1 vectors were treated with DNA methyltransferase inhibitor 5′-aza (Fig. S7a). To determine the role of SP1 in the regulation of FHL1 expression, we disrupted SP1 expression by siRNAs after knockdown of IMUP in BxPC-3 and SW1990 cells. The results indicated that FHL1 was inhibited by SP1 siRNAs after IMUP knockdown (Fig. S7b).

### FHL1 regulates cell-cycle protein kinases by interacting with CHK1/CDC25A/14–3-3ξ

**Fig. 7:**
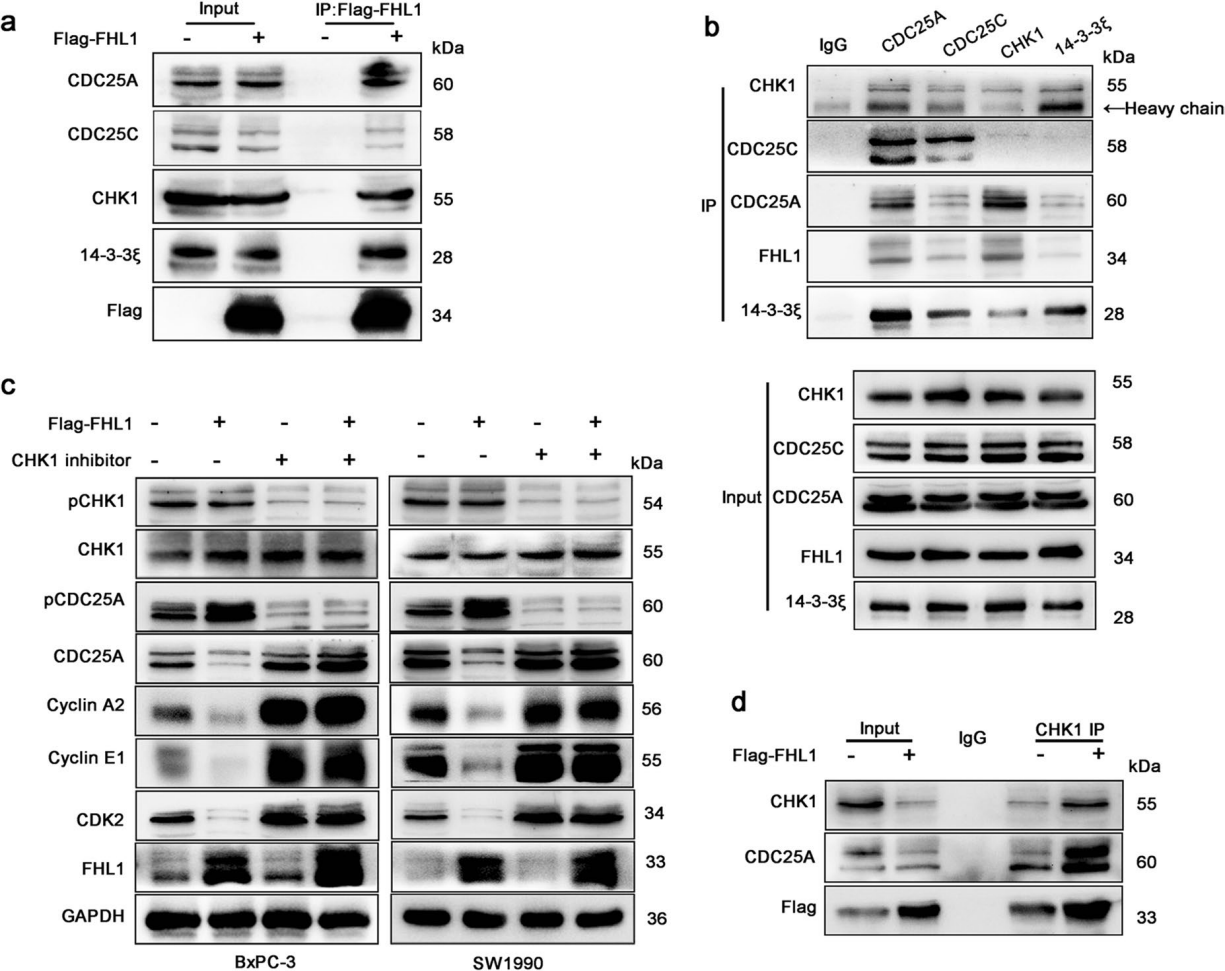
FHL1 interacts with CHK1/CDC25A/14–3-3ξ and promotes the phosphorylation of CDC25A via CHK1.** a** WB analysis of input and anti-Flag IP derived from BxPC-3 cells transfected with Flag-FHL1 vectors. **b** Endogenous proteins from BxPC-3 were immunoprecipitated using anti-CDC25A, CDC25C, CHK1, 14–3-3ξ antibody, or rabbit IgG as a negative control. **c** WB analysis of BxPC-3 and SW1990 cells treated with control vectors, Flag-FHL1 vectors, or/and treated with 25 μmol CHK1 inhibitor (GDC-0575 analog). **d** BxPC-3 cells were treated with control vectors or Flag-FHL1 vectors. WB analysis of IP by anti-CHK1 or IgG

IP was performed to explore the mechanisms by which FHL1 regulates cell-cycle arrest. After flag-tagged FHL1 vectors were transfected into BxPC-3 cells, FHL1-binding proteins were analyzed by LC/MS (Fig. S4b, Table S6). Multiple subtypes of 14–3-3 proteins have been identified as candidate-binding proteins, which are related to cell-cycle regulation (Gardino and Yaffe, 2011). Co-IP was used to validate the interaction between FHL1 and 14–3-3 proteins. We also verified the binding of FHL1 with CDC25A and CHK1 proteins, which reportedly interact with FHL1 in other cancer cells (Xu et al. 2017). The results showed that Flag-FHL1 interacted with 14–3-3ξ, CDC25A, CDC25C, and CHK1 (Fig. 7a). The interactions between FHL1 and 14–3-3ξ, CDC25A, CDC25C, and CHK1 were determined to be direct as endogenous 14–3-3ξ, CDC25A, CDC25C, and CHK1 bound to FHL1 (Fig. 7b).

CDC25 subtypes, including CDC25A, CDC25B, and CDC25C, play critical roles in the regulation of cell-cycle checkpoints (Donzelli and Draetta, 2003). Among them, phosphorylation of CDC25A by CHK1 generally contributes to cell-cycle arrest in the S phase (Zhao et al. 2002). FHL1 may regulate CDC25A activity by forming a protein complex with CHK1. Thus, we further investigated whether FHL1 regulated the phosphorylation of CDC25A and found that FHL1 overexpression promoted CDC25A degradation by phosphorylation, thereby inhibiting the protein levels of cyclin A2, cyclin E1, and CDK2 (Fig. 7c). However, FHL1 did not phosphorylate CDC25A directly, as the phosphorylation events were observed to significantly decrease following treatment of cells with a CHK1 inhibitor, which inhibited CHK1 phosphorylation (Fig. 7c). Moreover, overexpression of FHL1 increased the interaction between CDC25A and CHK1 (Fig. 7d). These results indicated that FHL1 increased CHK1-mediated phosphorylation of CDC25A.

**Fig. 8:**
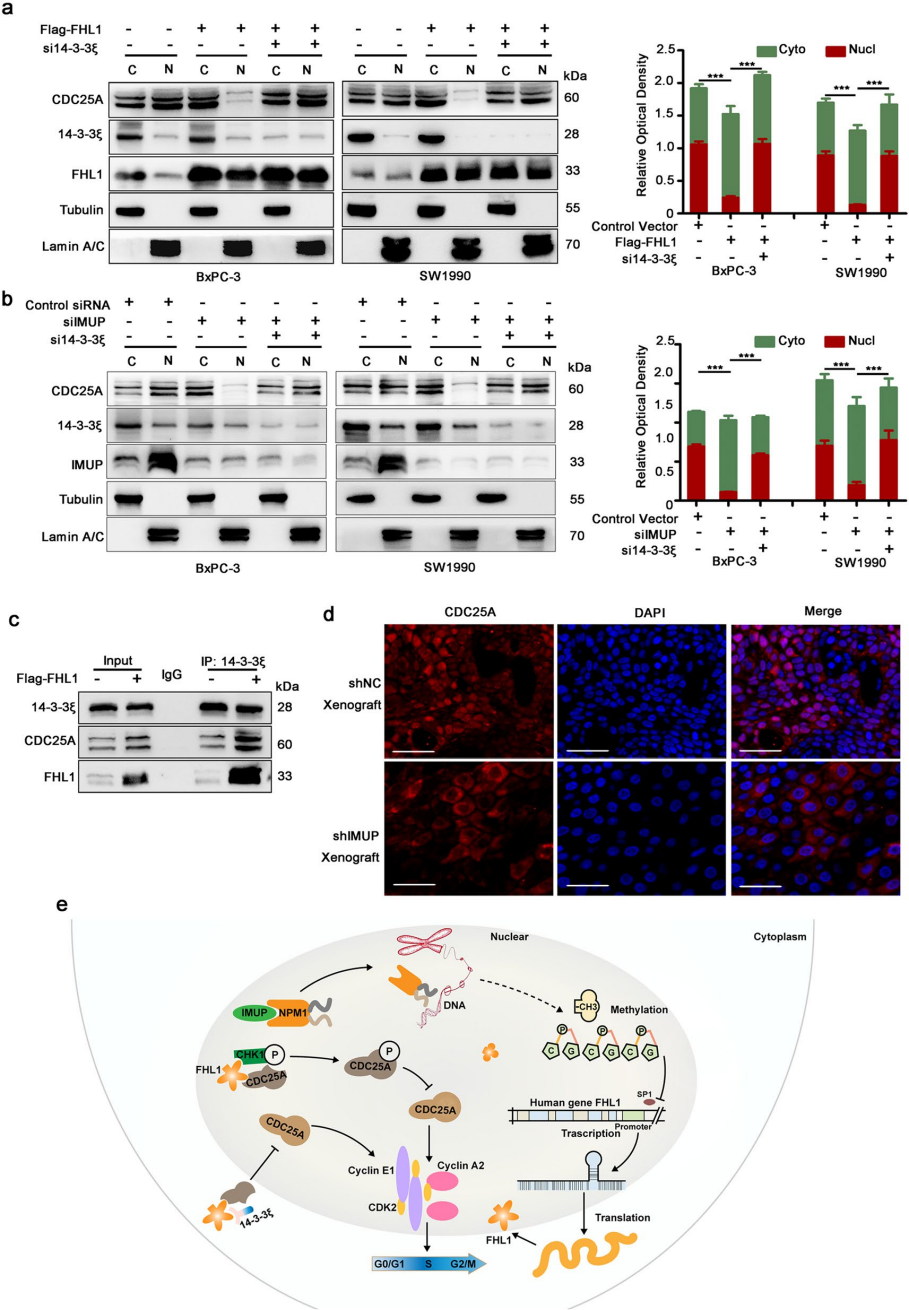
FHL1 causes CDC25A to become sequestered in the cytoplasm via binding to 14–3-3ξ.** a** BxPC-3 and SW1990 cells were treated with control vectors, Flag-FHL1 vectors, or co-transfected with Flag-FHL1 vectors and 14–3-3ξ siRNAs. Nuclear-cytoplasmic isolated proteins were used for WB analysis. C, cytoplasm; N, nucleus. **b** BxPC-3 and SW1990 cells were treated with control siRNAs, IMUP-siRNAs, or co-transfected with IMUP siRNAs and 14–3-3ξ siRNAs. Nuclear-cytoplasmic isolated proteins were used for WB analysis. Right panels show the densitometric analysis of CDC25A distributed in the cytoplasm or nucleus in three independent experiments. Statistical differences (the ratio of nucleus to cytoplasm) were analyzed by unpaired Student’s *t* test. ***P < 0.0001. **c** BxPC-3 cells were transfected with control vectors or Flag-FHL1 vectors. WB analysis of IP by anti-14–3-3ξ or IgG. **d** Representative immunofluorescence analysis of xenograft mouse tumors infected with control shRNA and IMUP-sh1. Cells were stained with anti-CDC25A (red). Nuclei are stained with DAPI (blue). Scale bars: 100 μm. **e** Schematic of the mechanism underlying IMUP-regulated S phase progression through NPM1/FHL1-mediated cell-cycle kinase protein activation. IMUP enhances the stability of NPM1 by direct binding. NPM1 indirectly facilitates promoter CpG island methylation of *FHL1* and inhibits the transcription of *FHL1*. FHL1 promotes the phosphorylation of CDC25A by CHK1 and sequesters CDC25A in the cytoplasm by forming CDC25A/14–3-3ξ complexes

It has been reported that 14–3-3ξ binds to CDC25A and prevents CDC25A from activating cell-cycle kinase in HeLa cells (Kohama et al. 2019). Binding between 14–3-3ξ and CDC25A in the cytoplasm inhibited CDC25A activity in cell-cycle progression (Al-Matouq et al. 2017). Further, we determined whether FHL1 affected CDC25A location. WB analysis of nuclear-cytoplasmic isolated protein showed that FHL1 overexpression increased the cytoplasmic distribution of CDC25A in BxPC-3 and SW1990 cells. However, 14–3-3ξ knockdown via siRNA neutralized the modulatory effect of FHL1 on CDC25A localization (Fig. 8a). We also verified that CDC25A was blocked in the cytoplasm by *IMUP* knockdown and that 14–3-3ξ siRNA could restore this effect (Fig. 8b). The mechanism underlying CDC25A distribution regulation by FHL1 was that FHL1 overexpression facilitated the interaction between 14–3-3ξ and CDC25A (Fig. 8c). In addition, immunofluorescence of xenograft tumors showed that CDC25A was transferred from the nucleus to the cytoplasm after IMUP knockdown (Fig. 8d). This should be attributed to the upregulation of FHL1 induced by IMUP depletion. These results suggest that FHL1 causes localization of CDC25A in the cytoplasm by forming a complex with 14–3-3ξ.

## Discussion

Sustaining proliferation signaling is a crucial mechanism in malignant tumors (Hanahan and Weinberg, 2011). Chemoradiation therapies aim to inhibit the proliferation of cancers by inducing DNA damage and cell-cycle delay (Wang et al. 2018a, b, c). However, only 4% of PDAC patients achieve a complete pathological response after chemoradiation therapy (Gillen et al. 2010). Cell cycle alteration is known to play a vital role in radiation resistance (Buckley et al. 2020). Here, we found that IMUP is significantly upregulated in PDAC tissues and is correlated with poor patient prognosis. In addition, we demonstrated that IMUP promotes proliferation and tumorigenicity of PDAC cells both in vitro and in vivo. However, the mechanisms underlying IMUP regulation in PDAC are poorly understood.

IMUP is reportedly associated with cell proliferation (Jeon et al. 2013) and cell-cycle regulation (Kim et al. 2000). Our results verified that IMUP depletion leads to S phase arrest in PDAC cells and decrease of cell-cycle related proteins, including cyclin A2, cyclin E1, and CDK2. CDC25A is a pivotal phosphatase for the activation of cyclin E/A-CDK2 kinase complexes and is a key S phase-promoting enzyme (Bartek et al. 2004). Hu et al. reported that hypericin-induced CDC25A inhibition led to S phase arrest and apoptosis by decreasing the CDK2/cyclin A complex in colorectal cancer (Hu et al. 2021). Sviderskiy et al. also determined the function of the ATR/CHK1/CDC25A/CDK2 DNA damage response axis in basal-like breast cancer (Sviderskiy et al. 2020). Another trigger cell-cycle checkpoint protein, CHK1, which is primarily activated in the S and G2 phases, can induce cell-cycle arrest by phosphorylating CDC25A in response to DNA damage (Rahnamay Farnood et al. 2021). Moreover, it was reported that the interaction of CDC25A with 14–3-3ξ inhibits cell-cycle progression in HeLa (Chen et al. 2003) and skin cancer cells (Holmes et al. 2020).

We examined the mechanisms by which IMUP regulates cell-cycle delay in PDAC and found FHL1 to be a significant contributor to this mechanism. FHL1 has been reported as a tumor suppressor in many cancers. However, although it contributes to the suppression of tumor growth and metastasis, recent studies have shown that FHL1 increases chemoradiotherapy resistance in some cancers (Asada et al. 2013; Xu et al. 2017; Ji et al. 2015; Zhou et al. 2018). Thus, some researchers considered that FHL1 may have promoting effects in cancer (Wei and Zhang, 2020). Nevertheless, the fact that FHL1 induces cell-cycle arrest was confirmed (Niu et al. 2012; Wong et al. 2010). Ren et al. also found that FHL1 can inhibit tumor progression of tongue squamous cell carcinoma by G1/S arrest (Ren et al. 2015). Similarly, we found that *IMUP* knockdown promotes FHL1 expression. Subsequently, FHL1 inhibits PDAC cell proliferation and tumorigenicity by inducing cell-cycle arrest.

FHL1 has been confirmed as a fragile tumor suppressor gene on chromosome X that is readily epigenetically silenced by DNA methylation (Asada et al. 2013). In fact, FHL1 is suppressed by promoter methylation in many cancers (Wang et al. 2014). Therefore, we hypothesized that IMUP decreases the transcription of *FHL1* via DNA methylation. Notably, IMUP has been identified as a nuclear protein (Kim et al. 2000). Moreover, another highly expressed nucleolus phosphoprotein, NPM1, was found to directly interact with IMUP in our study. Meanwhile, we also found that IMUP-depletion decreases the protein level of NPM1. Previous studies have shown that NPM1 plays a crucial role in cell-cycle progression and transcription regulation and promotes DNA replication in the S phase (Karimi Dermani et al. 2021; Qin et al. 2020). Nuclear NPM1 makes a significant contribution to cell-cycle progression from the S to G2 phase (Lim and Wang, 2006). Many studies have examined the mechanisms underlying NPM1 and have reported that NPM1 is closely related to DNA methylation (Wang et al. 2020; Wang et al. 2018a, b, c). Our pyrosequencing results revealed a high level of *FHL1* promoter methylation in PDAC cells in comparison with IMUP-depleted cells. This indicated that *IMUP* knockdown decreases DNA methylation of FHL1 and promotes binding of transcription factor SP1 to the *FHL1* promoter. However, NPM1 overexpression restores the methylation and, thus, inhibits *FHL1* transcription. Therefore, the results suggested that IMUP induces FHL1 methylation through NPM1.

We also demonstrated that FHL1 directly binds to CHK1 and CDC25A and promotes CDC25A phosphorylation. Degradation of CDC25A further leads to inhibition of the cyclin A2/E1-CDK2 complex and S-phase arrest. In addition, FHL1 promotes the interaction between CDC25A and 14–3-3ξ, which further inhibits the function of CDC25A in the nucleus by sequestering it in the cytoplasm. Furthermore, we found that FHL1 interacts with CDC25C, which was confirmed as an important factor in cancer development (Donzelli and Draetta, 2003). However, whether FHL1 regulates the progression of PDAC through CDC25C remains to be confirmed.

The IMUP/NPM1/FHL1-mediated CDC25A-CHK1-14–3-3ξ activity represents a novel insight into cell-cycle checkpoints (Fig. 8e). As such, an IMUP inhibitor or NPM1-induced-methylation blocker may serve as a novel therapeutic drug candidate for PDAC. In addition, the inhibition of IMUP may enhance chemotherapeutic effects in PDAC patients through cell-cycle arrests. However, this study has two major limitations that must be investigated further. First, the specific mechanism by which IMUP regulates NPM1 levels remains unclear. Second, more research is required to elucidate the detailed mechanism underlying NPM1-induced methylation of the *FHL1* promoter.

In summary, these novel findings regarding IMUP/NPM1/FHL1-mediated regulation of cell-cycle checkpoints may provide potential therapeutic targets for PDAC. To advance these findings, we will explore the mechanisms regulated by IMUP in more detail in future research. In addition, we will examine the effect of IMUP inhibition on PDAC chemotherapy.

### Supplementary Information

Below is the link to the electronic supplementary material.

Supplementary file1 (PDF 754 KB)

Supplementary file2 (PDF 180 KB)

## Data Availability

All data in this study are available from the corresponding author.

## Abbreviations

CDC25: Cell division cycle 25
CDK: Cyclin-dependent kinases
CHK1: Cycle checkpoint kinase 1
DEGs: Differentially expressed genes
FHL1: Four-and-a-half LIM domain protein 1
IMUP: Immortalization upregulated protein
NCGs: Negatively correlated genes
NPM1: Nucleophosmin
PDAC: Pancreatic ductal adenocarcinoma
SRGs: Survival-related genes
TCGA: The Cancer Genome Atlas
