# Supplementary material for: Immortalization-upregulated protein promotes pancreatic cancer progression by regulating NPM1/FHL1-mediated cell-cycle-checkpoint protein activity
Source: Cell Biol Toxicol. 2022 Feb 10;39(5):2069–87. doi: 10.1007/s10565-022-09695-4 (PMC10547647; doi:10.1007/s10565-022-09695-4)
Supplement: Supplementary file 1 — Supplementary file1 (PDF 754 KB) [file 10565_2022_9695_MOESM1_ESM.pdf]

Supplementary figures and supplementary figure legends

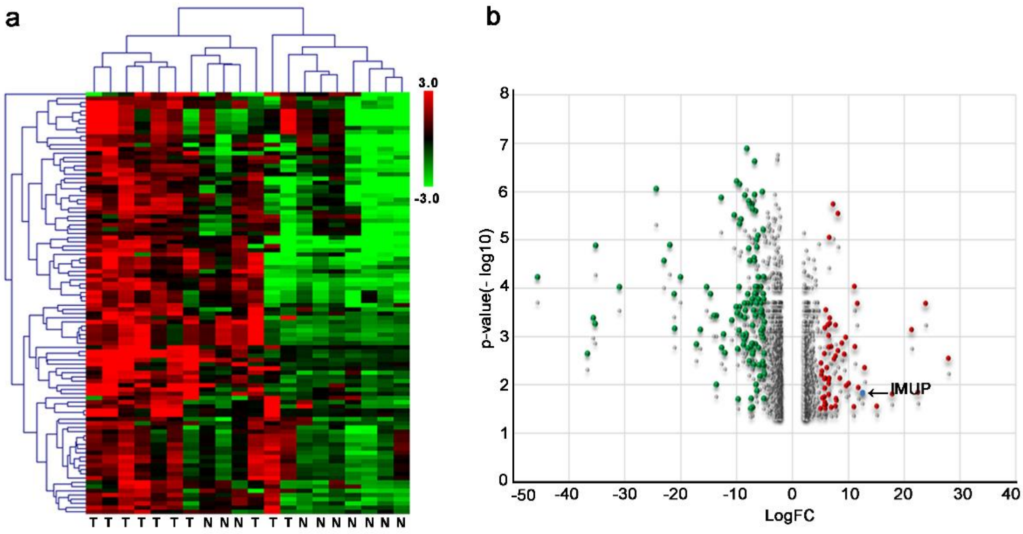

Fig. S1 Transcriptomics of tumor and adjacent normal tissues of ten paired primary PDAC patients by microarray scanning of GeneChip. (a) Heatmap and (b) volcano plot of differentially expressed genes with fold change ≥ 5. T, tumor tissues; N, adjacent normal tissues; LogFC, log (fold change). Black arrow indicates that IMUP is upregulated in tumor tissues (b).

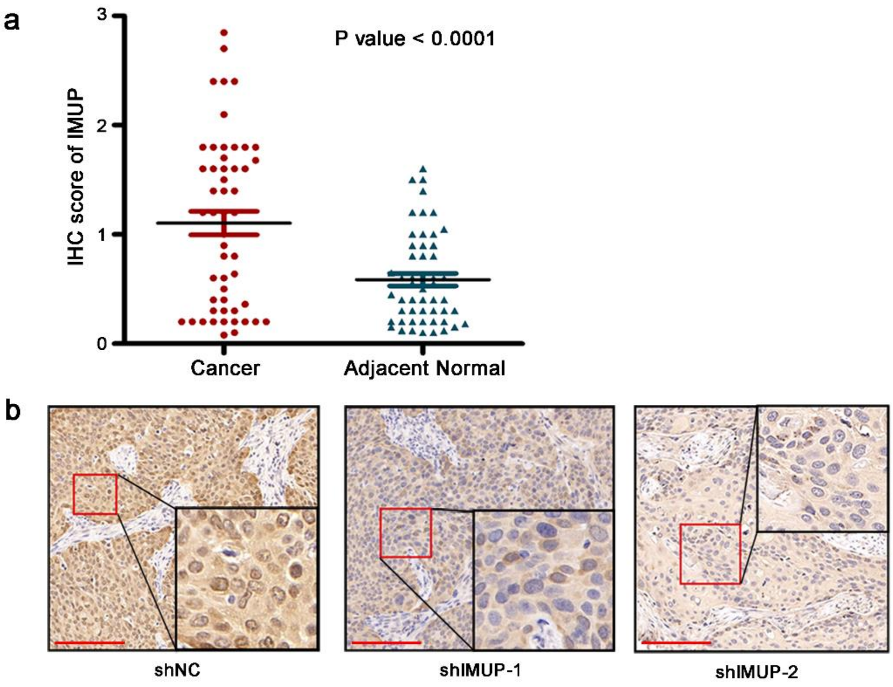

Fig. S2 Immunohistochemistry (IHC) by anti-IMUP. (a) IHC scores of cancer tissues and adjacent normal tissues from 52 PDAC patients. Data are analyzed by paired Student's *t*-test, $P < 0.0001$. (b)

Representative IHC of xenograft tumors transfected with shNC, IMUP-shRNA1 and shRNA2 by anti-IMUP.

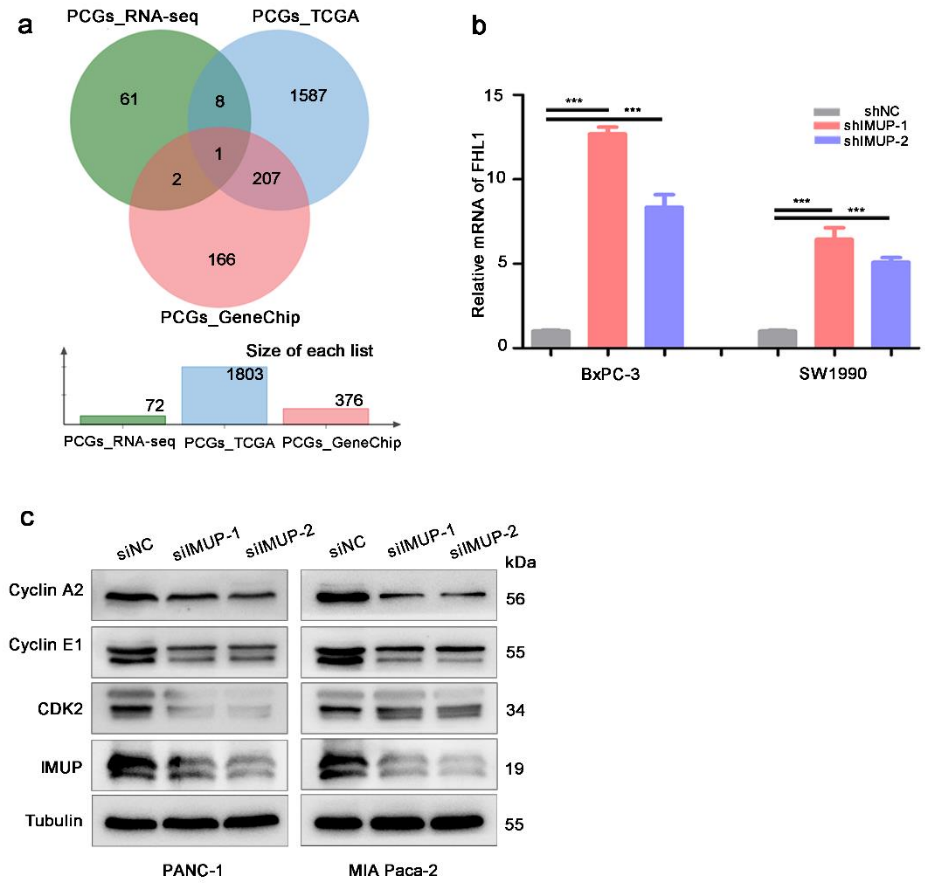

Fig. S3 (a) The intersection of PCGs from GeneChip, RNA-seq (fold-change > 2) and the TCGA database provided wingless-type MMTV integration site family member 10A (WNT10A). (b) BxPC-3 and SW1990 cells were treated with IMUP shNC, sh1, or sh2. RT-qPCR was used to detect *FHL1* mRNA level. Data were analyzed by unpaired Student's *t*-test, *** $P < 0.0001$. (c) WB analysis of PANC- and MIA Paca-2 cells transfected with IMUP-siRNA1 or siRNA2. Tubulin were used as a loading control.

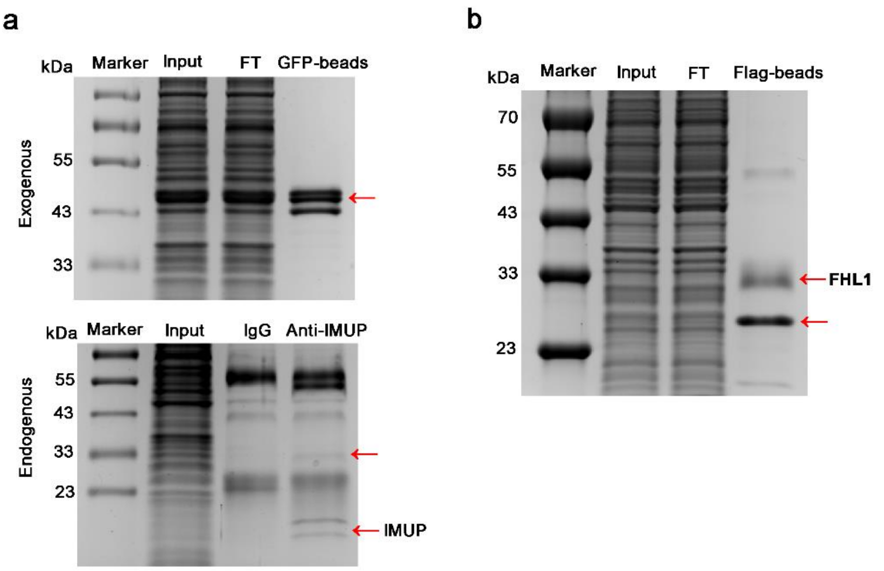

Fig. S4 IP complexes processed via LC/MS. (a) BxPC-3 cells treated with GFP-IMUP vectors were used for exogenous IP by anti-GFP (up). Cell lysates extracted from BxPC-3 cells were used for endogenous IP by anti-IMUP. (b) Cell lysates extracted from BxPC-3 cells treated with Flag-FHL1 vectors were immunoprecipitated by anti-Flag.

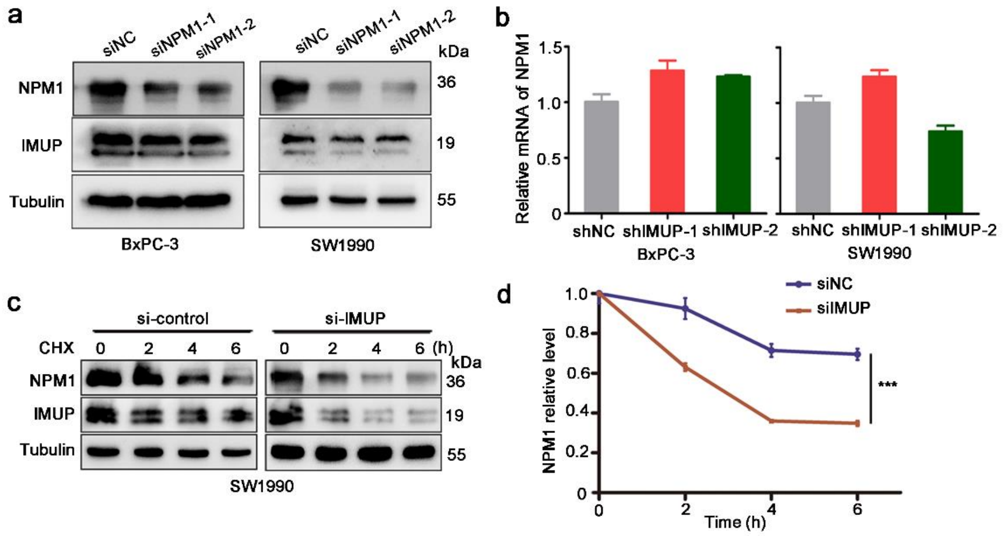

Fig. S5 IMUP enhances the stability of NPM1 protein. (a) WB analysis of BxPC-3 and SW1990 cells treated with siNC, NPM1-siRNA1 or siRNA2. (b) RT-qPCR analysis of NPM1 expression in BxPC-3 and SW1990 cells treated with shNC, IMUP-shRNA1 or shRNA2. * $P < 0.05$. (c) SW1990 cells infected with IMUP siRNAs or control siRNAs were treated with 100 μg/mL CHX at the indicated time. Proteins were analyzed with anti-IMUP, anti-NPM1 and anti-tubulin. (d) The densitometric quantitation of NPM1 protein at indicated time was normalized by Tubulin. Statistical differences were analyzed by two-tailed Student's *t*-test. *** $P < 0.0001$.

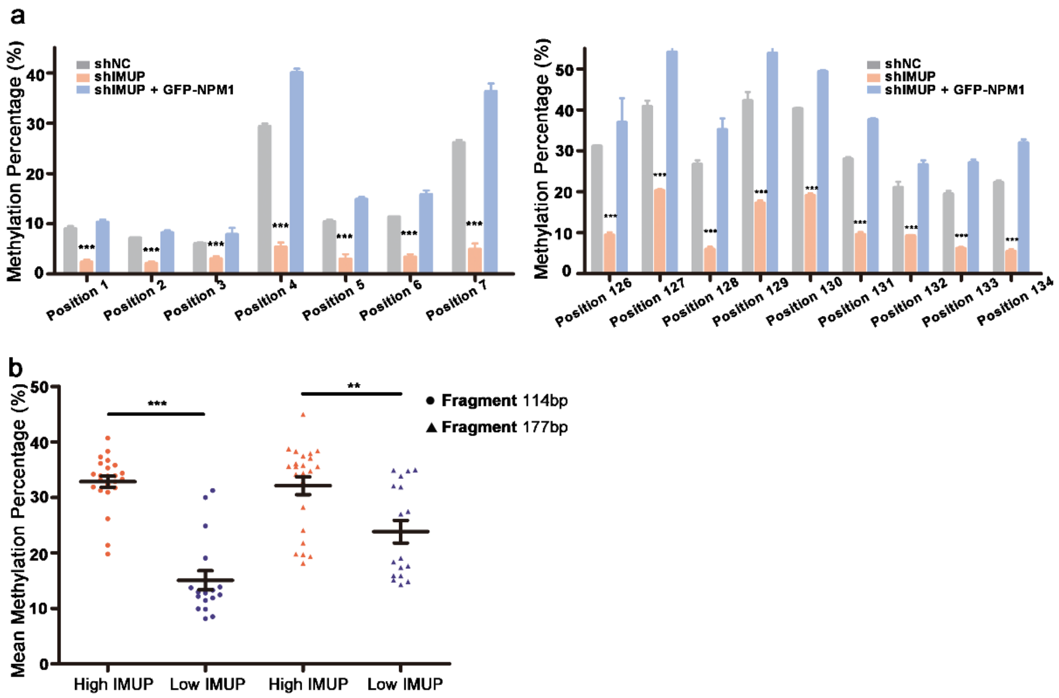

Fig. S6 The methylation status of FHL1 promoter in SW1990 cells and human tissues. (a) DNA was collected from SW1990 cells transfected with control shRNAs, IMUP-shRNAs, or co-transfected with IMUP-shRNAs and NPM1 vectors. The methylation percentage of *FHL1* promoter CpG islands. Fragment 1 (left histogram) and fragment 2 (right histogram). Statistical differences were analyzed using two-way ANOVA test. *** $P < 0.0001$. (b) The mean methylation level in fragment 114bp and 177bp of FHL1 promoter was verified in patients with high IMUP expression (n = 23) and low IMUP expression (n = 17). Statistical differences were analyzed by two-tailed Student's *t*-test. *** $P < 0.0001$, ** $P < 0.001$.

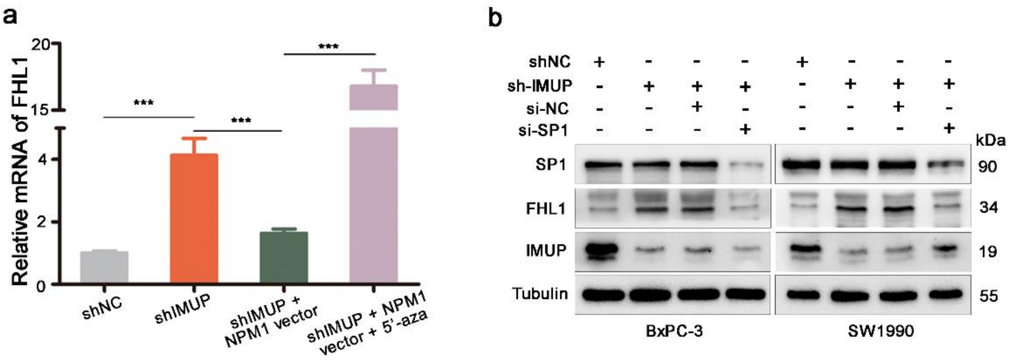

Fig. S7 IMUP regulates *FHL1* mRNA expression through NPM1-mediated DNA methylation and transcription factor SP1. (a) RT-qPCR analysis of *FHL1* mRNA expression in cells treated with shNC, shIMUP, or co-transfected with shIMUP and GFP-NPM1 vectors cells with or without 5'-aza-treatment. Data were analyzed by unpaired Student's *t*-test, *** $P < 0.0001$. (b) WB analysis after SP1 expression was downregulated using siRNAs in BxPC-3 and SW1990 cells transfected with IMUP siRNAs.
