# Supplementary material for: Immortalization-upregulated protein promotes pancreatic cancer progression by regulating NPM1/FHL1-mediated cell-cycle-checkpoint protein activity
Source: Cell Biol Toxicol. 2022 Feb 10;39(5):2069–87. doi: 10.1007/s10565-022-09695-4 (PMC10547647; doi:10.1007/s10565-022-09695-4)
Supplement: Supplementary file 2 — Supplementary file2 (PDF 180 KB) [file 10565_2022_9695_MOESM2_ESM.pdf]

Supplementary Table S1. Target sequences of shRNAs or siRNAs.

**Gene**	**Target sequence (5'→3')**
IMUP-sh1	CCAGCGATTCGGACACGGA
IMUP-sh2	AGCTCAGTCCCCACAAAGT
IMUP-sh3	CCTGGACAGGGCTCATTAA
IMUP-si1	GGAGTTCGACCTGGGAGCA
IMUP-si2	CGGGTCCAAAGCAAGGACA
IMUP-si3	TCGGACACGGATGTGAAGT
FHL1_sh	CTTGGCCAATGAGACCTTT
FHL1_si	GGGAAGAAGTATGTGCAAA
14-3-3ξ-si	AAACCATTACAACGAAGTCCCTCCC
NPM1-si1	TTCCTCCACAGCTACTAAG
NPM1-si2	GGACAAGAATCCTTCAAGA
SP1-si	GCCAATAGCTACTCAACTA

Supplementary Table S2. Antibodies used in Western blotting, immunoprecipitation, and liquid chromatography tandem mass spectrometry.

**Antibody**	**Lot No.**	**Company**
IMUP	ab228823	Abcam (Cambridge, UK)
IMUP	ab228821	Abcam (Cambridge, UK)
pCDC25A	ab156574	Abcam (Cambridge, UK)
pCHK1	ab79758	Abcam (Cambridge, UK)
CDK2	AF6237	Affinity Biosciences (OH, USA)
Cyclin A2	BF683	Cell Signaling Technology (Danvers, MA, USA)
Cyclin E1	HE12	Cell Signaling Technology (Danvers, MA, USA)
FHL1	10991-1-AP	Proteintech (Wuhan, China)
CDC25A	55031-1-AP	Proteintech (Wuhan, China)
CHK1	25887-1-AP	Proteintech (Wuhan, China)
CDC25C	16485-1-AP	Proteintech (Wuhan, China)
14-3-3ξ	11648-2-AP	Proteintech (Wuhan, China)
NPM1	10306-1-AP	Proteintech (Wuhan, China)
GFP-Trap® Magnetic Agarose	gtma-100	Proteintech (Wuhan, China)
Pierce™ anti-DYKDDDDK Magnetic Agarose	A36798	Abcam (Cambridge, UK)

Supplementary Table S3. The primer sequences for RT-qPCR.

**Primer**	**Forward 5'→3'**	**Reverse 5'→3'**
IMUP	GTCCGGGTCCAAAGCAAGG	GTGGGACTTCACATCCGTGT
FHL1	CGCTGTGGAGGACCAGTATTAC	TAGTCGTGCCAGGATTGTCCTT
NPM1	GGAGGTGGTAGCAAGGTTCC	TTCACTGGCGCTTTTTCTTCA
β-actin	CCAACCGCGAGAAGATGA	CCAGAGGCGTACAGGGATAG
FHL1 promoter site-1	GCACAGGCTTTATCCAAATTTGAT	AGCCCTCGCCATGTGCCAAGCT
FHL1 promoter site-2	AGTGACGTTAAAACGCTCTGGA	CTTCGGGGCCCACGCCGTTT
FHL1 promoter upstream	CATTGTTTTGGAAGTGGAGTAT	AGCACGAGGAAAACGGCCTTC
ACTIN promoter	TCGAGCCATAAAAGGCAACT	CTTCCTCAATCTCGCTCTCG

Supplementary Table S4. Univariate and multivariate cox regression analysis of potential prognostic predictors for PDAC patients.

	p	**HR**	**95%CI**
**Univariate analysis**			
Age	0.970	0.999	0.956-1.044
Gender	0.139	0.769	0.544-1.089
T status	0.800	0.940	0.585-1.511
N status	0.029*	2.074	1.077-3.995
AJCC stage	0.002**	2.649	1.443-4.861
IMUP	0.001**	3.638	1.699-7.789
**Multivariate analysis#**			
IMUP (high expression group vs. low expression group)	0.001**	3.695	1.660-8.225

Multivariate cox regression analysis was adjusted by gender, age, T status, N status, AJCC stage using Forward Stepwise (Conditional LR) method. *p<0.05, **p<0.01, ***p<0.001.

Supplementary Table S5. LC/MS showed the IMUP-binding proteins.

Genes	Molecular Weight （KDa）	Mean Protein Score[#]
NPM1	32.55	266.60
IMUP	10.89	171.21
ALB	69.32	127.07
ACTB	41.71	108.36
BRIX1	41.37	98.51
H1-2	21.35	86.45
FLG2	247.93	75.54
DCD	11.28	45.71
NAXA2	38.58	39.33

[#]Mean protein scores are the mean of GFP-beads LC/MS results and endogenous LC/MS results.

Supplementary Table S6. LC/MS showed FHL1-binding proteins.

Accession	Protein names	Gene names	MW [kDa]	Protein score	Sequence coverage (%)
Q13642	Four and a half LIM domains protein 1	FHL1	36.24	202.64	15.17
P27348	14-3-3 protein theta	YWHAQ	27.75	135.10	20.00
P63104	14-3-3 protein zeta/delta	YWHAZ	27.73	124.05	16.73
P28066	Proteasome subunit alpha type-5	PSMA5	26.39	122.84	20.33
O14818	Proteasome subunit alpha type-7	PSMA7	27.87	107.08	14.52
P31946	14-3-3 protein beta/alpha	YWHAB	28.07	106.71	19.92
P25789	Proteasome subunit alpha type-4	PSMA4	29.47	84.69	7.28
P05109	Protein S100-A8	S100A8	10.83	80.73	20.43
Q15517	Corneodesmosin	CDSN	51.49	69.08	5.10
Q02413	Desmoglein-1	DSG1	113.68	59.80	6.20
P62258	14-3-3 protein epsilon	YWHAE	29.16	48.27	7.06
